# Perspectives of Canadian Healthcare and Harm Reduction Workers on Mobile Overdose Response Services: A Qualitative Study

**DOI:** 10.1177/29767342241237169

**Published:** 2024-03-25

**Authors:** Navid Sedaghat, Boogyung Seo, Nathan Rider, William Rioux, S. Monty Ghosh

**Affiliations:** 1 Department of Medicine, Faculty of Medicine and Dentistry, University of Alberta, Edmonton, AB, Canada; 2 Cumming School of Medicine, University of Calgary, Calgary, AB, Canada; 3 Department of Internal Medicine, Faculty of Medicine and Dentistry, University of Alberta, Edmonton, AB, Canada

**Keywords:** overdose, supervised consumption sites, mobile overdose response, services, healthcare providers, harm reduction providers, opioid toxicity

## Abstract

**Background:**

Supervised consumption sites (SCS) are an evidence-based intervention proven effective for preventing drug overdose deaths. Obstacles to accessing SCS include stigma, limited hours of operation, concerns about policing, and limited geographic availability. Mobile overdose response services (MORS) are novel technologies that provide virtual supervised consumption to help reduce the risk of fatal overdoses, especially for those who use alone. MORS can take various forms, such as phone-based hotlines and mobile apps. The aim of this article is to assess the perceptions of MORS among healthcare and harm reduction staff to determine if they would be comfortable educating clients about these services.

**Methods:**

Twenty-two healthcare and harm reduction staff were recruited from Canada using convenience, snowball, and purposive sampling techniques to complete semistructured interviews. Inductive thematic analysis informed by grounded theory was used to identify main themes and subthemes.

**Results:**

Four themes were identified: (1) increasing MORS awareness among healthcare providers was seen as useful; (2) MORS might lessen the burden of drug overdoses on the healthcare system but could also increase ambulance callouts; (3) MORS would benefit from certain improvements such as providing harm reduction resources and other supports; and (4) MORS are viewed as supplements for harm reduction, but SCS were preferred.

**Conclusions:**

This research provides valuable perspectives from healthcare and harm reduction workers to understand their perception of MORS and identifies key areas of potential improvement. Practical initiatives to improve MORS implementation outcomes exist.

**Highlights**

- This is the first study seeking healthcare and harm reduction providers’ perspectives on mobile overdose response services.
- Participants highlighted increasing awareness of mobile overdose response services among healthcare providers.
- Mobile overdose response services can reduce the burden on the healthcare system while also having potential downsides, such as increasing ambulance callouts.
- Supervised consumption sites were preferred over mobile overdose response services but can improve by providing resources to other supporting bodies.

## Introduction

The harm reduction model of substance management gained significant momentum between 1970 and 1980 to curb transmission of infectious diseases such as human immunodeficiency virus and hepatitis B.^1^ It is now a widely recognized patient-centered approach to reduce the harms associated with substance use without imposing or necessitating abstinence.^2,3^

Supervised consumption sites (SCS) have since been recognized as the “gold standard” for reducing harms associated with substance use by administering various services, including but not limited, to naloxone administration/overdose response, drug checking, and referrals to long-term health and social services.^4-6^ However, these efforts continue to be plagued by numerous logistical, social, and political challenges (eg, stigma, altercations with police/law enforcement, long wait times, and limited operating hours) that hinder people seeking harm reduction and promoting safer substance use.^7-11^ The most recent data reported by Health Canada reveal that almost 4000 lives were lost to opioid toxicity between January and June 2023.^12^ The alarming number of fatal overdoses across the country suggest a need for more comprehensive and innovative measures to combat the opioid epidemic.

Mobile overdose response services (MORS) are novel technologies that have emerged to address the unmet needs of the current opioid crisis. They are specially designed to provide virtual supervised consumption for clients who cannot access an SCS.^13,14^ These digital interventions are considered valuable adjuncts to SCS and have been adopted in numerous countries such as Canada, the United States, and the United Kingdom.^14,15^ MORS are available in various formats but often come in forms of hotlines and mobile apps (Table 1). In Canada, the National Overdose Response Service (NORS) hotline and Brave app are available nationally to provide virtual connection to a service operator who monitors the client’s call during their substance use.^16^ Typically, an individual with lived or living experience of substance use is the line operator, initiating an emergency response (consisting of either emergency medical services [EMS] or a community-based responses) if someone becomes unresponsive. Timer-based apps, such as Lifeguard^17^ (available in British Columbia and Northern Ontario) and Digital Overdose Response System (DORS^18^; available in Alberta), prompt users to reset a timer at intervals during their session. Both these apps operate similarly and if the user fails to reset the timer, the app triggers an emergency response. Other technologies operate on a similar principle.^13^

**Table 1.** Different MORS Types, Their Availability, and Operation.^19^

Location	Service	Type of service	Established	How does it operate?
Canada only	NORS	Hotline	2020	Operators monitor peers using substances through a phone call. If no response is received, emergency services are dispatched to the peer’s location
	DORS (Alberta only)	App-based	2021	The app starts an emergency alarm if the user does not check in within 60 seconds, after which emergency services are dispatched
	Lifeguard (British Columbia and Ontario only)	App-based	2020	A 50-second alarm is triggered, which must be turned off by the user. EMS is dispatched after 75 seconds
	Better App	App	2020	Guardian angels are sent if the emergency alarm is not shut off by the user
	iKeepr	App	2021	Guardian angels are sent if the emergency alarm is not shut off by the user
United States only	Never Use Alone	Hotline	2019	Operators monitor peers using substances through a phone call. If no response is received, emergency services are dispatched to the peer’s location
	The Canary—Prevent Overdose app	App	2018	Users are monitored through movement, respiration, and response to prompts. An emergency alert is triggered if no response is received
	Unity Philly (Philadelphia only)	App	2019	Users are connected to others who are trained and carrying naloxone in the event of an emergency
	Naxos Neighbors	App		Guardian angels are sent if the emergency alarm is not shut off by the user
Both Canada and the United States	BeSafe Brave	App/hotline	2020	If the caller becomes unresponsive, an operator will contact the users’ emergency contacts or EMS

Abbreviations: MORS, mobile overdose response services; NORS, National Overdose Response Service; DORS, Digital Overdose Response System; EMS, emergency medical services.

With increasing reliance on telemedicine to facilitate provider-patient relationships, there is a critical need to understand how healthcare providers, harm reduction workers, and other key interest groups perceive MORS to identify the strengths and challenges of these emerging technologies. These services are crucial in reaching out to users. Hence, it is vital to comprehend how healthcare providers and harm reduction staff view them.

## Methods

The Consolidated Criteria for Reporting Qualitative Research (COREQ) checklist was used to guide the reporting of the results. The study adhered to ethical guidelines by the Tri-Council Policy Statement for Ethical Conduct for Research Involving Humans and the Helsinki Declaration.^19^

### Selection of Participants

Semistructured interviews of healthcare providers and harm reduction workers were conducted between November 2021 and April 2022. A mix of snowball, convenience, and purposive sampling methods were used to recruit participants through existing networks of MORS staff and the research investigators. All participants had to meet the following inclusion criteria: 18 years of age or older, reside in Canada at the time of study, have adequate English proficiency, and have access to a mobile device.

### Interviews

The semistructured interview guide was created by MORS operators, People Who Use Substances (PWUS), and the research team. All participants provided verbal consent and received concise background information about MORS before beginning each interview. Interviews were conducted by third-party consultants (SJ and LA) via phone or video conferencing platforms (eg, Zoom) and ranged in duration from 20 to 60 minutes. Participants had the right to withdraw at any time. Mental health support was available if required by the participants. Interviews were recorded using *TapeACall* and transcribed manually by a professional transcription service.

### Analysis of Data

Transcripts underwent coding and thematic analysis using *Dedoose* software. Masters-level evaluators proficient in qualitative methods (SJ and LA) performed all coding with oversight from the principal investigator (MG). The analysis adhered to the frameworks of Braun and Clarke and Glaser and Strauss to guide the thematic analysis informed by grounded theory.^20,21^ The evaluators co-reviewed the initial 3 transcripts to establish thematic consistency and developed a shared codebook. Each evaluator independently coded subsequent transcripts and regularly reviewed each other’s work. Coding responsibilities were evenly distributed between the evaluators, with consistent cross-validation. Any coding discrepancies were resolved through discussions among the evaluators and consultation with a project manager (LA) and principal investigator (MG) to achieve consensus. Following the initial coding phase, evaluators, the project manager (LA), and the principal investigator (MG) assessed thematic saturation across all participants. Interviews continued until this saturation point was reached by identifying no novel themes. Data triangulation with healthcare providers, frontline harm reduction workers, and theme validation were undertaken as additional measures.

## Results

Interviewees (n = 22) consisted of physicians, nurses, and a variety of other allied health professions from various disciplines, such as emergency medicine, addiction medicine, and psychiatry. The demographics of these individuals are shown in Table 2.

**Theme 1:** MORS could be integrated into various care pathways to increase its awareness among healthcare providers.

**Table 2.** Demographics of the Participants Who Work in the Healthcare and Harm Reduction Field.

Healthcare roles	Province
Registered nurse harm reduction	Alberta
Primary care physician (n = 1)	Alberta
Nurse educator (n = 2)	Alberta, British Columbia
Registered nurse (n = 2)	Alberta
Healthcare manager (n = 3)	Alberta
Healthcare manager (n = 1)	Nova Scotia
Community harm reduction worker (n = 3)	Alberta
Community harm reduction worker (n = 1)	Yukon
Community harm reduction worker (n = 1)	Newfoundland and Labrador
Community addiction worker (n = 1)	Saskatchewan
Peer operators (n = 2)	Alberta
Peer operators (n = 2)	Ontario
Peer operators (n = 2)	Nova Scotia

Interviewees highlighted how general awareness of conventional harm reduction services and strategies may be lacking among healthcare workers, let alone the understanding of virtual harm reduction technologies like MORS. They suggested that the key step toward achieving a wider adoption of MORS is by integrating advertising campaigns for MORS into various outpatient care settings. Some respondents deemed that public health agencies and larger governing bodies should bear more responsibility in advocating for these services.

*[It] has the potential to be quite impactful, again, because a lot of the people that we [healthcare providers] serve don’t have access to any other means of support while they’re using drugs. I think that in general, [SCS] and [MORS] are not nearly widespread or normalized enough in our system*. (Community harm reduction worker, Alberta)

*I would be asking the health authorities in different provinces to promote more—whether it’s posters*. (MORS operator, Ontario)

In certain instances, participants viewed MORS as counterproductive to their clients’ treatment and recovery goals. There were concerns that MORS would promote unregulated substance use and further the cycle of addiction.

*At one level I think it’s making it easier for people to continue to make that choice to use, rather than getting to the point of this isn’t comfortable.* (Community addiction worker, Saskatchewan)

*I believe they support keeping people in active addiction. Rather than encouraging them to look at an alternative*. (Community addiction worker, Saskatchewan)

More evidence-based education and resources regarding harm reduction were seen as necessary to resolve the misconception that these services encourage dangerous and riskier substance use. Respondents believed that these efforts would create a more inclusive and safer atmosphere where PWUS are open to seeking assistance from healthcare professionals. In addition, the harm reduction providers needed to understand the nature of substance use in their area.

*Yeah, like [SCS] where there’s a lot of misinformation and stigma, like I would say a lot of the [healthcare] staff are weary to promote something like that because I feel like that’s enabling. So I think a lot of the barriers can be addressed by providing more education about [SCS]*. (Acute care registered nurse, Alberta)

*Well, it’s one more tool in the toolbox that we can give people to stay alive. You know, once I give people the resource, it’s up to them to use it, right, but if that resource didn’t exist, then that’s one less thing that people have to keep them safe.* (MORS operator, Nova Scotia)

*[Its concerning that] Somebody who lives in Manitoba is talking to somebody in Alberta and not understanding the culture, the drug supply, in that city.* (Primary care healthcare provider, British Columbia)

**Theme 2:** There remains an ambivalent attitude about whether MORS can alleviate the strain on the emergency services and healthcare system.

Some respondents believed that MORS could reduce the overall financial burden on the healthcare system by reducing the frequency of ambulance callouts and emergency department (ED) visits.

*I think it would be, likely, a huge relief to the health system. I think our healthcare system is certainly overworked right now, so an approach like this to keep somebody out of a hospital.* (Rural harm reduction worker, Nova Scotia)

On the contrary, some interviewees remained skeptical regarding whether MORS can address the ambulance shortage, as EMS could still be dispatched in the event of an overdose. They worried about adding to the burden of first responders and overworked emergency services.

*But I think even if somebody is overdosing like an ambulance is still dispatched. And yes that person may not go to the hospital, but you are still taking away from like the EMS*. (HIV clinic worker, Alberta)

*So I think it can go either way. It can either be a nice support to the services that are already available, and lessen the burden on, but it could also tax it even more if it’s not well thought out and run.* (Primary care physician, Alberta)

**Theme 3:** MORS should bridge access to other harm reduction tools, substance use treatment, and mental health services.

Beyond overdose prevention, the ability of hotline-based MORS to connect their clients to additional mental health resources was perceived as beneficial. One respondent emphasized the limited capacity of the acute care settings to handle parasuicidal behaviors and other psychological crises. One suggestion was to recruit more staff who were not only familiar with harm reduction but also emotional support, de-escalation, and other social services (eg, foodbanks). Some respondents highlighted the advantage of having healthcare providers offering necessary medical information on the line.

*Just having an experienced clinician on the other end of the phone I think is important.* (Acute care registered nurse, Alberta)

*People call in when they’re lonely—and where would you refer them to, where they can talk to someone? Or if they have severe, severe mental health illnesses . . . and there’s a crisis and this is a repeated incident, where?* (Rural harm reduction provider, Alberta)

*If the person on the other line was a nurse that has harm reduction experience I think would be really helpful.* (Acute care registered nurse, Alberta)

On the other hand, one MORS staff emphasized that the operators should not impose addiction treatments or create unwarranted pressure to utilize other services to clients, which could discourage them from seeking MORS in the future. Some also advised against offering mental health support through hotline-based MORS as it will duplicate the types of services already available through other organizations and could overwhelm the system’s capacity to monitor and assist individuals for supervised consumption. Some even suggested that MORS could be integrated into existing addiction phone-based services.

*They provide a safe space for them to use it, create connectivity in terms of providing them with emotional support and peer support. But they also offer access to [. . .] mental health support, addiction support, counselling, other things*. (MORS operator, Ontario)

*There’s mental health hotlines that people can call in [. . .] if someone is having to wait for an operator to use then it becomes a—they might just get frustrated, hang up and use on their own and then something happens.* (Community harm reduction provider, Alberta)

**Theme 4:** While MORS are acceptable adjunct services to address the current gaps in harm reduction services, SCS are viewed as the most comprehensive mode of supporting PWUS.

Overall, respondents believed that MORS are necessary services to appropriately meet the needs of the current overdose crisis, especially for individuals who use substances alone. They speculated that there would be a higher rate of preventable overdose-related deaths if such services were unavailable.

*I think ultimately we would be looking at saving lives. [. . .] I think ultimately the outcome could be more lives lost and lost unnecessarily, right?* (Rural registered nurse, Alberta)

*This provides a bit more reassurance that they might stay alive, it provides an additional support, you know, just in case. The more supports you can provide, the better.* (Community addiction worker, Saskatchewan)

There was also a consensus, however, that MORS should not be treated as a substitute for SCS. For instance, some healthcare and harm reduction workers were reluctant to recommend MORS to their patients as they believed that virtual services were less equipped to respond promptly to an overdose than the physical sites.

*You know, obviously if you’re on a site there’s probably some basic first aid available, and you have someone who can react to you quite quickly. If you’re alone, [. . .] you just have to wait for someone to come. So it still seems like it could possibly delay care.* (Primary care physician, Alberta)

*If EMS services don’t arrive on time, or if the community response that’s initiated with a family member, a friend, doesn’t arrive on time. If those are not available, then that could be a huge problem for the client, and we won’t be able to support them*. (MORS operator, Nova Scotia)

In addition, it was noted that PWUS may build more robust and meaningful relationships with SCS staff than a virtual service. Respondents believed that engagement with harm reduction workers in-person fosters a unique environment for PWUS to share their concerns about their substance use and other healthcare needs.

*I feel like if I were to show up at an [SCS], I would be developing a relationship with another human being that I can see and talk to every day, or however often I go. Which I think you’re probably more likely to develop those relationships quicker if you are seeing somebody in person.* (MORS operator, Ontario)

Despite the view that SCS are ideal, most participants said they would recommend MORS to others. They suggested that MORS would benefit by creating a “one-stop shop” environment.

*Before [MORS] there was not really no other support service out there to help support this population. So this really does provide an opportunity to help support this group that has difficulties accessing harm reduction supports.* (MORS operator, Ontario)

*Definitely, I think it just gives a bit more reassurance to providers. It also helps family members, I think. I’ve had lots of family members who told me that they wish they had this service when the [ir] child was alive, because they would have made use of this. Almost all the moms that I’ve spoken to, and the parents, had all lost children at home, because they were using alone. And so almost every one of them.* (Community harm reduction provider, Newfoundland and Labrador)

*I think it would do a really good job of filling a gap, especially in a community where [SCS] don’t exist.* (Registered nurse, Alberta)

*I think just thinking of things like accessibility and lack of [SCS] in our own community. And I would say it was one of those things that I knew existed or had heard of, but would not have been something top of mind for me to think in offering to my patients. But now I will more so, and maybe it’s something in my role as an educator that I educate staff on the, hey, this exists. So we don’t have these types of services in our community, but we do have other options.* (Acute care registered nurse, Alberta)

## Discussion

This is the first study to examine the perceptions of healthcare providers and harm reduction workers toward the role of MORS in Canada. The following key themes were identified: (1) the need to improve awareness of MORS in clinical settings; (2) its implications on the EMS and the healthcare system; (3) the opportunity to connect clients to further addiction treatment, mental health supports, and other medical services; and (4) how MORS can effectively complement the goals of SCS.

Despite the alarming prevalence of opioid toxicity deaths and other growing drug-related harms,^12^ the perception toward harm reduction services remains variable across Canada.^22-24^ While there is a positive trend among healthcare systems to acknowledge substance use disorder (SUD) as a complex biopsychosocial illness,^25,26^ many medical professionals continue to hold stigmatized and inaccurate beliefs that could negatively affect patient care and their health outcomes.^27^ A 2013 systematic review of studies conducted in Australia, the United States, Canada, and Ireland found that healthcare workers often perceived patients with SUD as manipulative, violent, and poorly motivated.^27,28^ This was especially true for those in disciplines not related to addiction medicine.^9,20^ Indeed, stigma and implicit bias from healthcare providers can serve as barriers to promoting MORS to patients who may benefit from these services.^29^ During the interviews, some concerns related to how MORS could enable substance use, despite evidence in the current literature suggesting that harm reduction interventions like SCS do not promote substance use or other criminal activities.^30^ The authors believe there is a need for more comprehensive and up-to-date educational materials for healthcare providers, regardless of their discipline, to take a more trauma-informed approach when working with vulnerable populations, which can include more frequent interactions with PWUS during medical training.^31^

As with any new technology, implementation of novel technologies requires persistent effort to increase awareness.^32^ Participants suggested utilizing existing public health campaigns to raise awareness of MORS. Few health authorities in Canada, such as Alberta Health Services, have begun advertising DORS app on naloxone kits.^33^ These kits contain a QR code that directs clients to download the mobile app.^33^

PWUS are estimated to access the hospital and emergency services 7 to 8 times more frequently than the general population for substance use and mental health-related problems.^28^ There was a mixed sentiment among the respondents regarding the implications of MORS on EMS and other healthcare sectors. Some believed that MORS could lessen the financial costs related to ambulance callouts and ED visits. For instance, mobile apps like DORS and Lifeguard already contain resource tabs to inform individuals about the appropriate services.^17,18^ Operators from hotline-based MORS like NORS have begun to offer mental health support and psychosis de-escalation to clients.^34^ However, a few respondents indicated that MORS could increase the number of ambulance calls and potentially overwhelm the current capacity of the healthcare system. Overall, the authors believe that MORS can be leveraged to refer PWUS to more formal and reliable mental health supports, which may deter unnecessary involvement of emergency services. Nonetheless, there is a paucity of research examining the impact of MORS on the healthcare system, though research is ongoing.^14^

Although participants identified the opportunity to improve access to mental health, addiction treatment, and other medical assistance through MORS, there were also concerns that this could undermine its main objectives to support PWUS for supervised consumption. NORS is already known to provide peer support and referrals to other hotlines for their clients, such as the Kids Help Phone and the Crisis Center, designed specifically for mental health support. The optimal degree of integration between MORS and existing agencies has yet to be determined. From the authors’ point of view, forming partnerships with existing harm reduction organizations and mimicking their “one-stop shop” model might be beneficial. Such an endeavor may be overwhelming for MORS as they currently stand, but adequate oversight and collaboration with existing agencies may be needed to prevent unnecessary overlap between services.

While telemedicine rapidly gained popularity during the COVID-19 pandemic,^35^ it has also shown to take away the human aspect of medicine due to a lack of shared physical space.^36^ One 2023 qualitative study of primary care patients with chronic medical conditions described telemedicine as “psychologically different than being at a doctor’s office” and “harder to establish empathy.”^36^ One of the limitations of MORS noted by participants is the challenge of building a therapeutic relationship with the operators. While this may be possible in a virtual care setting, SCS facilitates a more suitable environment for establishing social connections through in-person communication.^37^ In addition, automated app-based services could not support such relationships at all.^38^ Study participants revealed how hotline operators should refer clients to necessary social services and support to mitigate this shortcoming. Similarly, app-based services should explore innovative ways to provide users with a comprehensive resource list.^38^

Despite the perceived utility of MORS among many respondents, many supported SCS due to their robust evidence regarding its effectiveness and safety.^4,7,8^ Recently published data from NORS reported 77 overdose incidents that did not lead to fatalities or prolonged hospitalization.^39^ Unity Philly, a mobile application in the United States designed to facilitate rapid naloxone administration through engagement with bystanders, likewise demonstrated a 96% success rate in reversing overdose, with only one reported fatality.^37^ Nonetheless, some interviewees voiced concerns regarding response times for overdose when using MORS. This was an important consideration given that Canadian provinces currently face delays in response times due to a shortage of ambulance and EMS personnel.^40-42^ However, MORS are primarily aimed at a subpopulation of PWUS who are unable to access alternative means of supervised consumption due to reasons such as their route of substance consumption not being supported (eg, inhalation), long commute to the nearest SCS, and substance use outside of SCS/Overdose Prevention Site (OPS) operating hours.^43-46^ The authors wish to highlight that while MORS complements the existing harm reduction services, it should not be considered a substitute for SCS. We believe that providing a clearer explanation of the objectives of MORS and its intended clients will encourage healthcare workers to be more inclined to recommend these services to their patients.

### Strengths and Limitations

Our study managed to represent the diverse perspectives of key interest groups from various disciplines and geographical regions across Canada (urban and rural). This aspect is essential as the nature of substance use and the provision of harm reduction services may differ depending on the jurisdiction that the interviewees reside in. One limitation, however, is the sampling bias due to snowball, convenience, and purposive recruitment of participants based on known and existing networks. Future explorations of this study should aim to elucidate the opinions of first responders, such as paramedics and police officers, toward MORS.

### Conclusion

This study examined the perspectives of healthcare providers and harm reduction workers toward MORS. It was believed that increasing MORS awareness among healthcare workers would be beneficial. While MORS may help reduce the pressure on the healthcare system, there remains pitfalls to mitigate. Providing referrals to other harm reduction, mental health, and addiction treatment resources was also recommended to improve the utility of MORS. Although MORS can complement other harm reduction services, SCS were still preferred. The areas of strengths and weaknesses identified by these key interest groups will inform the future development and implementation of MORS.

## Supplemental Material

sj-docx-1-saj-10.1177_29767342241237169 – Supplemental material for Perspectives of Canadian Healthcare and Harm Reduction Workers on Mobile Overdose Response Services: A Qualitative Study Supplemental material, sj-docx-1-saj-10.1177_29767342241237169 for Perspectives of Canadian Healthcare and Harm Reduction Workers on Mobile Overdose Response Services: A Qualitative Study by Navid Sedaghat, Boogyung Seo, Nathan Rider, William Rioux and S. Monty Ghosh in Substance Use & Addiction Journal
