# Supplementary material for: Perspectives of Canadian Healthcare and Harm Reduction Workers on Mobile Overdose Response Services: A Qualitative Study
Source: Subst Use Addctn J. 2024 Mar 25;45(3):506–14. doi: 10.1177/29767342241237169 (PMC13021089; doi:10.1177/29767342241237169)
Supplement: sj-docx-1-saj-10.1177_29767342241237169 – Supplemental material for Perspectives of Canadian Healthcare and Harm Reduction Workers on Mobile Overdose Response Services: A Qualitative Study [file sj-docx-1-saj-10.1177_29767342241237169.docx]

| **INTERVIEW GUIDE** | |
| --- | --- |
| **QUESTION** | **PROBE** |
| **Awareness** |  |
| Had you heard of virtual supervised consumption services before us contacting you? | **If yes,** do you recall where or how you first heard of virtual consumption sites?  Can you tell me what you know about VSCS? |
| Do you think there is high awareness of VSCS among those who choose to use substances? | **If yes**, why? |
| I am going to briefly explain three examples of virtual supervised consumption services and then ask you what you think about them. The first is a phone hotline known as the National Overdose Response Services (NORS). Anyone can call the number for NORS and someone will answer the hotline who uses substances, have used substances in the past, or have another personal experience with drug use. This service is confidential. That operator will ask for the caller’s location and what substances they are using. That operator will stay on the phone with the person using substances (the caller) and call for help if the caller becomes unresponsive. The operator will check in with the caller every 30 seconds to 1 minute. If the caller does not respond the co-created safety plan will be enacted (i.e., a close contact may be engaged to support the client in an overdose situation as provided during the phone call and emergency services are deployed). Callers will also have the option of accessing additional substance use-related and health service referrals from the line should they require it.  The BRAVE app is available to anyone in North America through the Google Play and Apple store and allows people who use alone to request remote supervision and anonymous overdose support. This virtual safe consumption space has person-to-person connection in which overdoses are detected and a community-led response is initiated. The person uses the digital app to request support before they use. A suitable supporter is identified through the app; the supporter is then connected to monitor the caller over the phone. The caller and supporter set agreements and establish trust and the supporter remains on the line to make sure the caller is safe. If the supporter suspects an overdose, they start the response process.  The DORS app is available to people in the greater Edmonton and Calgary area and can be downloaded from the Google Play and Apple store. The app allows people to confirm their location and start a timer when they are ready to use a substance. Once the timer counts down to 30 seconds an alarm is sounded that will increase in volume. The timer can be disabled or extended at any time by the app user. If the app user does not respond and the timer ends, an emergency alert is sent to a Response Centre. The Response Centre will try and call the app user to see if they require assistance. If the app user says they need assistance or they don’t answer, medical help is sent to their location. The DORS app also provides information on national and provincial addiction recovery supports and services. | |
| **Adoption** |  |
| **Health Care Professional**:  Do you know if any of your patients have ever used any type of VSCS before?  Do you know of anyone else who has used any type of VSCS before? | **If yes**, do you recall which ones?  **If yes**, do you know if the operator/app connected the patient with other resources in the community?  **If yes**, do you recall which ones?  **If yes,** were there differences in the nature of the calls you responded to between VSCS and supervised consumption sites? Were there any differences in response times? |
| Why do you think some patients choose to use VSCS?  Why do you think some patients choose to not use VSCS?  Are there any other barriers to people using VSC services? (e.g., worried about sharing their information)? | Would COVID-19 factor into their decision at all? How?  What other factors could play into their willingness to try?   - Difficulty accessing safe consumption sites? - Safety at safe consumption sites? - Privacy and anonymity of VSCS? - Discrimination? - Privacy and anonymity? - Being treated with dignity and respect? - Personal/cultural beliefs? |
| How do you think someone’s gender and cultural background may impact their acceptance of using VSC in phone based and app-based form? |  |
| Who do you think would benefit most from VSCS? | What setting would they be in? (e.g., private accommodation) |
| Have you had patients that have used supervised consumption sites? | If yes, have they discussed any differences in using supervised consumption sites and VSCS? E.g., response time for overdose? |
| **Acceptability** |  |
| Do you feel the service fits your needs as a health care or emergency professional?  Do you think VSCS are acceptable and/or useful?  Do you feel the service supports individuals in their substance use (or in harm reduction)?  Do you feel the service provides access to greater supports for individuals who use? | Why or why not? |
| Do/would you recommend VSCS to patients?  Do you feel like you have enough information about the service to recommend VSCS to patients? | Why or why not?  What other information or what would you need to have to make you feel more comfortable recommending VSCS to patients? |
| What are your thoughts on other existing harm reduction measures for people who use substances? | Is there anything VSCS can learn from them? |
| **Equity** | |
| Do you feel the VSC service allows for equitable (e.g., increased fair and unbiased) access to harm reduction supports? | In what ways is the service not equitable (e.g., fair or is biased) in its delivery of supports? |
| In what ways can the service delivery be more equitable (e.g., fair and unbiased) in settings where financial and human resources are low? |  |
| In what ways does the service remove or reinforce barriers to access care, especially for those who have mobility issues, mental health concerns, lack of access to Wi-Fi or phonelines? |  |
| In what ways does this service meet or not meet the needs of minority groups and populations? | In what ways is this service appropriate or not appropriate for both urban and/or rural populations?  In what ways does the service meet the needs of women?  In what ways does the service meet the needs of LGBTQ2S+?  In what ways does the service meet the needs of Indigenous Peoples?  In what ways does the service meet the needs of other minorities?  What can we do to ensure it is more appropriate for these groups?  Do you feel the service has been able to reach the intended targeted populations? |
| In what ways can we improve connectivity of the service with other resources in community? |  |
| **Appropriateness** | |
| Do you think virtual supervised consumption services could be used for supports not related to opioid use? | **If yes,** what kind of support and why?  Probe specifically for Methamphetamine concerns  Mental health support?  Addiction support? |
| What potential impacts would there be to the health system if it were to be used for supports beyond overdose response? | ED impact?  Inpatient impact?  Patient experience impact? |
| What types of processes would need to be developed if VSCS were used for supports not related to substance use or to be used in your community? | Communication processes?  Documentation processes?  Coordination of care processes? |
| Are there opportunities to access virtual supervised consumption services in other settings? | Hospitals?  Community-based settings? Where else?  What barriers would there be?  How would this help the person using or thinking about using substances?  The community?  The health system? |
|  |  |
| If virtual supervised consumption services didn’t exist, how would that impact you in your professional role? | Your family/friends?  Your community?  The health system? |
| What about on the flip-side, are there any disadvantages to virtual supervised consumption sites? | For people who use substances?  For community?  For health systems? |
| **Feasibility** | |
| What, if any, recommendations do you have for how to improve virtual supervised consumptions services for people who use substances? And the safety of PWUS? | Anything it should *start* doing?  Anything it should *stop* doing?  In what ways can we improve upon the safety of both the phone and app-based service? |
| What, if any, recommendations do you have for how to improve virtual supervised consumptions services for others and your community? | People who are contemplating using?  Other health care providers?  Others? |
| What, if any, recommendations do you have for how to successfully integrate virtual supervised consumption service in <acute care> or <primary care>? | What is needed to successfully implement VSC in settings such as community clinics/harm reduction/housing facilities? |
| In what ways do you feel the program needs to be expanded? |  |
| Is there anything else that I haven’t asked you about that you’d like to share? |  |
|  |  |
| Only a few more quick questions. The following questions will be used to help us understand if virtual supervised consumption services affect different people and groups in different ways. Please note no information that identifies you, including the following information, will be reported with any of the other information you provided in the report. Again, you do not have to answers any of the questions if you do not want to. | |

| Demographic Questions | |
| --- | --- |
| Questions | **Probe** |
| 1. What organization do you work for? (if a health care provider) what type of health care provider i.e., clinician, administrator? |  |
| 1. What is your role at the organization? |  |
| 1. Which type of health care setting do you work? | Acute Care  Primary Care  Emergency Services |
| 1. In which province or territory are you currently residing? |  |
| 1. Do you consider the place you currently live to be urban or rural? |  |
|  |  |

Thank you so much for your time. Please feel free to contact me if you think of anything else that you’d wish to include. Take care.
